# A Comparison between Primary and Secondary Flap Coverage in Ridge Preservation Procedures: A Pilot Randomized Controlled Clinical Trial

**DOI:** 10.1155/2019/7679319

**Published:** 2019-08-20

**Authors:** Majdi A. Aladmawy, Zuhair S. Natto, Bjorn Steffensen, Paul Levi, Wai Cheung, Matthew Finkelman, Yumi Ogata, Yong Hur

**Affiliations:** ^1^Department of Periodontology, Johns Hopkins Aramco Healthcare, Dhahran, Saudi Arabia; ^2^Department of Periodontology, Tufts University School of Dental Medicine, USA; ^3^Department of Dental Public Health, School of Dentistry, King Abdulaziz University, Jeddah, Saudi Arabia; ^4^Department of Public Health and Community Service, Tufts University School of Dental Medicine, USA

## Abstract

**Aims:**

To assess the bone dimensional changes after extraction and alveolar ridge preservation (ARP) using primary coverage (closed flap technique, CFT) or healing by secondary intention (open flap technique, OFT).

**Materials and Methods:**

Ten patients (split mouth design) were planned for extraction and ARP. All sites received ARP with freeze-dried bone allograft (FDBA) and nonresorbable membrane after extraction. Clinical standardized measurements were used to assess the dimensional alterations of the alveolar ridge.

**Results:**

All patients completed the study, and a total of 20 sites were randomized to CFT or OFT group. Center height (mean difference of 8.1 mm, SD =1.9 CFT, and 7.5 mm, SD= 1.8 OFT) and buccal height (mean difference of 0.8 mm, SD =1.0 CFT, and 0.3 mm, SD= 1.1 OFT) were significantly different within the same group. However, there was no statistically significant difference between groups. In the OFT group, the keratinized tissue width was higher and the pain VAS scores at 24 hours were lower compared with the CFT (*p *= 0.004 and p = 0.006, respectively).

**Conclusions:**

Leaving the flap open did not have any effects on the dimensional changes of bone height or width. However, there was a wider band of keratinized tissue and less pain with the CFT compared with the OFT. The study protocol was registered at ClinicalTrials.gov, Identifier NCT03136913.

## 1. Introduction

When a clinician treatment plans for a tooth extraction only, bony changes follow as a consequence. The changes might result in unfavorable bone resorption with diverse volumetric patterns, due to different responses from osteoclastic and osteoblastic activity around the extraction socket. This can have an impact when planning to restore the extracted site, which might cause aesthetic or functional concerns in the site planned for implant therapy [1–6].

It has been reported in the literature that more bone is resorbed on the buccal than on the lingual side [7]. The main cause for this loss is the lack of bony plate thickness on the buccal/facial site. This thin bony plate also increases the chance of buccal dehiscence when teeth are still present, a most commonly encountered problem in implant dentistry [8]. The occurrence of a dehiscence is comparable to the occurrence of a three-wall bone defect. The fewer the bony walls that exist, the less the chance to maintain a blot clot provided by the extraction socket itself. Subsequently, the use of membranes will increase the regeneration process in dehiscence defects [9, 10].

The majority of bone loss occurs in the first month after extraction [6]. The amount of bone loss in the first three years after tooth extraction varies around 40-60% [11, 12]. The technique to preserve the alveolar ridge volume by incorporating foreign materials into a human extraction socket was initially described in the mid-80s [13, 14]. The clinician aims to preserve or gain a sufficient width and height of bone when teeth are removed. By evaluating the width of the ridge, Schropp et al. found a reduction of the width by approximately 50% from 12 to 5.9 mm [6]. Two-thirds of the bone loss occurred during the first three months of healing. The percentage of bone-width reduction has been found to be larger in the molar regions than in the premolar regions, and in the mandible compared to the maxilla [15]. Between the three- and the six-month evaluation, only minor bone changes were observed [16, 17]. Equally, only minor bone changes were observed between the six- and the twelve-month evaluation [16, 17]. The maximum bone loss evaluated close to the adjacent teeth of the extraction socket after 12 months was found to be 1.2 mm. A mean vertical loss of 1 mm could be determined in this study [16, 17]. Tiefengraber et al. found in a prospective split mouth study with a low number of evaluated patients that much more horizontal bone width could be preserved after extraction, when only a Gore-Tex membrane was placed over the extraction socket. After approximately 6 weeks the guided bone regeneration (GBR) site lost 1.1 mm in the buccal-lingual dimension, while the control site had lost 3.2 mm in the horizontal dimension. The radiologic examination of the vertical bone loss did not show any difference in this particular study [18].

There are two techniques that are commonly used for extraction and alveolar ridge preservation (ARP), either to close the wound of the extracted tooth by primary closure (i.e., closed flap technique, CFT) or to leave the extraction socket wound heal by secondary intention (i.e., open flap technique, OFT) [17, 19]. Primary closure is the approximation of the flap edges into close intimate contact via sutures (covering the membrane) whereas healing by secondary intention leaves the flaps in their original location following extraction with the membrane exposed [20, 21]. Iasella et al. presented clinical success of the procedure when they left the membrane exposed without primary coverage. They found the sites treated with bone grafts and resorbable membrane had lower bone loss following extraction when compared to sites without bone grafts and membrane [2].

On the other hand, Lekovic and coworkers reported on extraction and ARP with primary coverage. They found that the closed flap technique reduced the alveolar bone dimensional changes following extraction when compared with the sites without site preservation [3].

There are a few studies comparing the open and closed flap techniques in site preservation procedures [22–26]. However, the current literature is lacking in evidence to indicate which technique is superior. The limitations of those studies are (A) having one continuous surgical flap on open and closed techniques [26] and (B) lack of split mouth design utilizing a nonresorbable membrane [22, 23]. Moreover, surgical techniques in ARP procedures performed with d-PTFE (high-density polytetrafluoroethylene) membrane have not been thoroughly studied.

Therefore, the current pilot trial was conducted to compare the outcomes of the two surgical techniques, open and closed flap in ARP, using clinical and pain/discomfort analysis. This study is considered as a pilot study because the data are lacking in information regarding the standard deviation and the use of nonresorbable membrane that compares both open flap technique and closed flap technique following extractions.

## 2. Materials and Methods

This study was a single center pilot study, randomized controlled clinical trial, split mouth designed. It was performed from January 2015 to January 2016. The study protocol was registered at ClinicalTrials.gov (NCT03136913) and approved by the Tufts Health Sciences Institutional Review Board, Boston, Massachusetts (#11441). Subjects were recruited from Tufts University dental clinics.

### 2.1. Participants

Patients with a bilateral extraction (canine to molar) sites of teeth located on the same arch and having a treatment plan of extraction and ARP were included in the study. Additional inclusion criteria included the following: at least 18 years of age, no medical or dental contraindications, existence of at least 3 intact walls and half of the fourth bony wall as determined by bone sounding, no pregnancy, and no smoking. A consultation visit was conducted to determine the subject's eligibility, and a written informed consent was obtained prior to enrollment. All subjects were enrolled in the study following completion of phase I periodontal therapy (e.g., oral hygiene instructions, scaling and root planning, and prophylaxis). Phase I therapy was performed for all patients until a plaque score of < 15% and bleeding score of <10% were achieved.

### 2.2. Clinical Measurements

**Figure 1:**
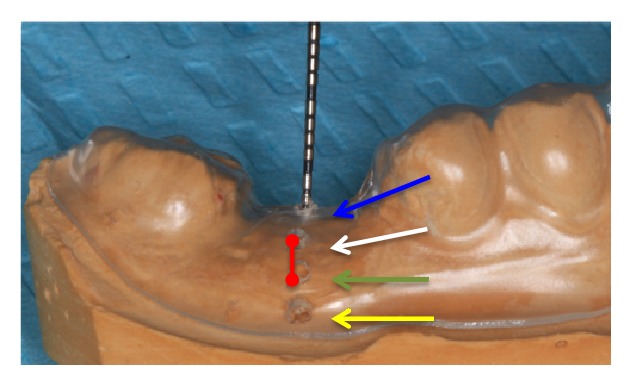
*Stent used to calculate crestal bone height and width*.* Blue arrow*: mid-socket bone height measurement (CH).* White arrow*: buccal bone height measurement (BH).* Green arrow*: width at 3 mm from crestal bone height (CW).* Yellow arrow*: width at 5 mm from crestal bone height (AW).* Red line*: keratinized tissue width (KTW).

Design and fabrication of standardized stents for clinical measurements have been described in detail in a previously published study [27]. Briefly, two impressions were taken for each patient. The stent had 4 standardized holes: mid-buccal area just above the buccal crestal bone height (BH), center of the extraction socket (CH), and width at 3mm (CW) and 5mm (AW) apical to the bone crest margin (Figure 1). The measurement of the bone width was done with a UNC-15 periodontal probe that passed through the hole until it came into contact with buccal bone. The following clinical parameters were recoded: plaque index (PI), bleeding index (BI), and keratinized tissue width (KTW). All these parameters were measured twice at baseline and 6 months.

All measurements were recorded by one calibrated examiner (MA). Intraexaminer calibration was carried out by 10 fellow residents at two different sites twice. All the measurements were approximated to the nearest 0.5 mm.

### 2.3. Surgical Procedure

The randomization was generated using the statistical package software (R Version 2.11.1) to determine which side was in CFT or OFT group. Atraumatic extraction using periotome instruments and extraction forceps was performed. For multirooted teeth, the roots were sectioned first before extraction, and then each root was extracted as a single rooted tooth. Attention was given to the maintenance of the buccal alveolar wall to minimize the amount of forces applied to buccal bone.

In the CFT group and after the lingual releasing incision, a buccal flap with two vertical releasing incisions and a trapezoidal shape flap were conducted at the closest line angles of the neighboring teeth mesially and distally. The incision was allowed for a full thickness flap and passed the mucogingival junction (MGJ). Then, a split thickness dissection was made apical to the MGJ to allow for coronal repositioning of the flap. In the OFT group, intrasulcular incisions were made and extended on the buccal and on the lingual sides to involve at least one tooth adjacent to the planned extraction tooth. The papilla and buccal attached gingiva were preserved and undermined to allow space for membrane adaptation.

Once all the measurements were done, both groups received freeze-dried bone allograft (FDBA, BioHorizons MinerOss™) after being hydrated, following manufacturer's instructions. A d-PTFE nonresorbable membrane (Osteogenics Biomedical Cytoplast™ TXT-200) was trimmed to the correct dimensional width of the alveolar ridge and extended 3 mm apical to the cementoenamel junction (CEJ), underneath the flap margins. After that, flaps were repositioned to cover the membrane in the CFT by the use of sutures (Ethicon Coated VICRYL®, polyglactin 910), while in the OFT group, the flaps were repositioned and the membrane was left exposed. Sutures were placed to stabilize the membrane in both groups.

At the end of the surgery, the participants were given a VAS pain questionnaire on a scale of 0-10 (with 0 being no pain at all and 10 being the worst possible pain). The subjects were asked to rate their pain at 24 hours and rate each side separately.

### 2.4. Postoperative Instructions

Patients received the following: amoxicillin (500 mg tid) for one week, chlorhexidine (3m Peridex™ Chlorhexidine Gluconate 0.12%, 1oz, bid) for 10 days, and ibuprofen (Pfizer Advil®, 600mg tid for three days) given to manage postsurgical discomfort and inflammation.

Another VAS pain questionnaire was given at 14-day follow-up to evaluate pain on each side of the surgical procedure separately at that time.

### 2.5. Sample Size and Statistical Analysis

No formal sample size calculation was performed because this is a pilot study. Ten subjects were recruited and completed the study. Descriptive statistics (e.g., means, standard deviations, median, and interquartile ranges) were computed for each group. The primary outcome was to evaluate the difference in alveolar bone height and width changes between groups. The Wilcoxon signed-rank test was used to assess statistical significance. P-values less than 0.05 were considered statistically significant. SPSS version 22 (IBM Inc., Armonk, NY, USA) was used in the analysis.

## 3. Results 

### 3.1. Demographics

Demographic characteristics were the same for both groups due to design of the study. Out of 10 patients, 8 were males and 2 were females. The average age was 56.4 years old (SD = 9.1), and the range was 46 to 71 years old (Table 1). The study had 1 canine, 6 premolars, and 3 molars paired to each other on the contralateral side (a total of 20 sites). Three patients had contiguous sites that needed extraction and ARP on each side.

### 3.2. Changes in Clinical Measurements

**Figure 2:**
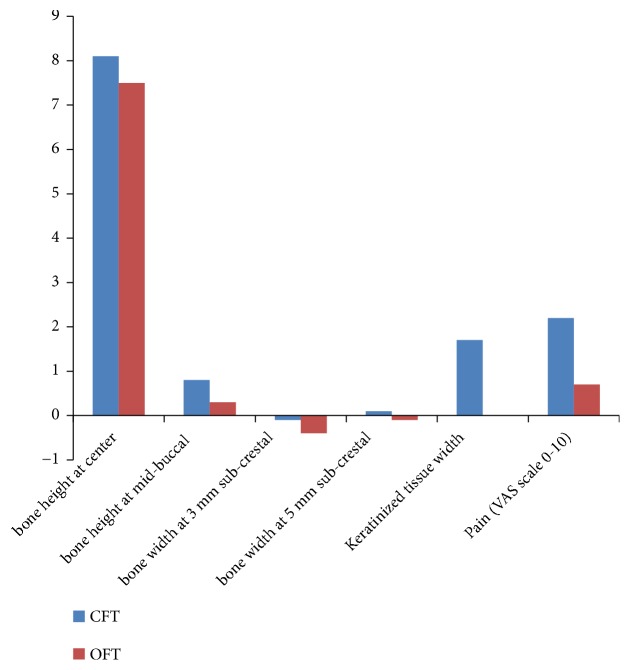
Net difference gain between baseline and 6 months (CFT: closed flap technique, OFT: open flap technique).

Table 2 shows the clinical measurements changes after 6 months of healing. There were no statistically significant differences in plaque index (PI) and bleeding index (BI) between baseline and 6 months for the subjects (*p* = 0.643 and* p* = 0.809, respectively) (Table 2 and Figure 2). There was a significant increase in center height when compared to the baseline within both groups (*p* = 0.005 for both groups) (Table 3 and Figure 2). CFT had a mean difference of 8.1 mm, SD=1.9, more than the OFT group (mean gain of 7.5 mm, SD=1.8). There were no statistically significant differences within the same group regarding BH and bone width at 3 mm and 6mm subcrestal to the mid-buccal surface. When comparing the bone gain difference in CH, BH, or bone width at 3 mm and 6mm subcrestal in CFT against OFT, there was no statistically significant difference between the groups (*p* = 0.389) (Table 3 and Figure 2).

#### 3.2.1. Changes in Keratinized Tissue Width (KTW) and VAS Pain Score

There was a statistically significant loss in keratinized tissue width within CFT group only of about 1.7 mm, SD= 0.6 mm. OFT had a significantly higher KTW at 6 months (mean of 3.4 mm, SD= 1.2, median of 3.5 mm, IQR= 2) compared to CFT (mean of 2 mm, SD= 0.9, median of 2 IQR= 1) (*p* = 0.011) (Table 3 and Figure 2).

OFT had a statistically significant lower pain score at 24 hours compared with CFT (a mean of 1.1 mm, SD= 0.5 OFT, and mean of 3 mm, SD= 0.8: CFT) (Table 3 and Figure 2). At 2 weeks following extraction, there was no statistically significant difference between CFT and OFT in the pain scale (*p* = 0.132). Both groups had a statistically significant reduction in pain when comparing 24 hours following surgery to 2 weeks following surgery (*p* = 0.006 and 0.008, respectively) (Table 3 and Figure 2).

## 4. Discussion

The current study evaluated two surgical techniques after extraction and ARP. Both sites had FDBA and a nonreinforced d-PTFE (high-density polytetrafluoroethylene) membrane. The present findings confirm that complete preservation of the ridge after tooth extraction is unlikely to be achievable and leaving the flap open (OFT) did not have any effects on the dimensional changes of bone width or height. It may have a positive effect on keratinized tissue width and postoperative pain compared with closing the extraction socket with a flap (CFT).

Several ARP techniques have reported some bone loss, even if extensive variations were used [2, 4]. Among the different regeneration techniques, the present study used corticocancellous FDBA substitute and a nonreinforced d-PTFE membrane. Two different surgical techniques were consequently implemented: complete coverage of the membrane (CFT) or a flapless procedure leaving the membrane exposed (OFT). Certainly there are several factors affecting dimensional alterations following tooth extraction. However, one factor which may play a significant role is the type surgical procedure performed: flap or flapless tooth extraction. Fickl et al. observed that, on a canine model, OFT group had a less bone loss compared with the CFT [28]. Conversely, Araujo and Lindhe [29] reported that raising a flap during extraction may had an effect on the short-term only. However, the difference between the two techniques was insignificant after 6 months.

In the current study, the two surgical procedures had similar results regarding bone changes when compared with each other. Both techniques had statistically significant difference in the center of the socket height (CH) within the same group. The CH will always be different and changed compared to the baseline in grafted or nongrafted sites [30]. There was an interesting finding in the present study in the mid-buccal socket wall height, that is, more bone height at 6 months compared with baseline in the CFT group.

In general, the vertical bone augmentation is more unpredictable compared with the horizontal bone augmentation [31, 32]. Several studies found that the use of grafting materials in socket spaces will minimize the bone loss in width and height compared with extraction only [27, 30, 33, 34]. However, it remains uncertain whether the successful outcome is influenced by the presence or absence of a primary wound closure [35, 36]. Aimetti et al. [37] who used a different bone graft, OFT and secondary closure techniques, reported a mean buccolingual width loss of 1.6 mm and height loss of 0.8 mm for the test group. Aimetti's report of remodeling was greater than the alveolar ridge height remodeling recorded in this paper, with a mean width loss of 0.1 mm in the CFT and 0.4 mm in the OFT.

The bone loss in the horizontal dimension at 3mm and 5mm apical to bone crest was probably due to the exposed nonresorbable d-PTFE membrane (0.4 mm at 3mm and 0.1 mm at 5mm). This might be related to the difficulty in maintaining the area free of inflammation especially at the membrane margins. Compressive forces due to the sutures during the early healing process appeared to be less in the OFT group than with in the CFT group (0.4 mm SD=1.6 mm). Moreover, the primary closure accomplished by the CFT procedure seemed to maintain the vertical dimension better than the OFT group on the buccal aspect. The buccal site is the one more likely to be exposed due to bone changes.

In the OFT procedure, the d-PTFE membrane was left intentionally exposed to the oral cavity, and sutures were used mainly to stabilize the membrane. The progress of secondary wound healing appeared to be slower than the primary healing of the CFT group, regardless of the presence of the two release incisions. Difficulties were encountered due to the need for regular plaque removal of the ARP with OFT that could have played a significant role in bone loss.

Some of the limitations of this study were the convenience sample which is not representative and the total number of teeth included in the study. However, this is a pilot study, because we did not find any similar study using the same combination of bone graft and membrane. There were three patients with paired contiguous extraction sites, which confound the outcomes. Literature found that contiguous teeth extraction led to more bone loss compared with a single tooth extraction [38]. Moreover, the measurements were done using a stent at standardized points to directly measure the bone in a linear location relative to the stent hole. These data provide information at those sites only, as it lacks information on the volume. Further studies and analysis of other variables, such as defect size, and remaining walls condition are essential to confirm the current findings.

## 5. Conclusion

Within this pilot study's limitations, the present findings confirm that complete preservation of the ridge after tooth extraction is unlikely to be achievable. It was found that leaving the flap open did not have any significant effects on center or buccal bone height, and 3mm or 5mm bone width. However, there was a wider band of keratinized tissue and less postoperative pain at 24 hours in the OFT compared with the CFT.

## Figures and Tables

**Table 1:** Summary of demographic data.

Variable	results
Age, Mean (SD)	56.4 (9.1)
Age Range	46 to 71
Male, n (%)	8(80)
Female, n (%)	2(20)
Paired Canine, n (%)	1(10)
Paired Premolar, n (%)	6(60)
Paired Molar, n (%)	3(30)
Contiguous extraction pairs, n (%)	3(30)

**Table 2:** Means and standard deviations for plaque and bleeding scores at baseline following phase 1 therapy and at 6 months after extraction and ridge preservation.

Variables	Mean±SD	Median (IQR)	*P*-Value
Plaque at baseline	16.2±2.2	16.5(3.3)	0.643
Plaque at 6 months	16.0±3.3	15.5(4.8)	
Bleeding at baseline	14.9±2.3	14.5(2.8)	0.809
Bleeding at 6 months	15.0±2.9	14.0(4.0)	

**Table 3:** Stent measurement at baseline and 6 months following extraction and ridge preservation.

	Variables	CFT		OFT		*p* value
		Mean±SD	Median (IQR)	Mean±SD	Median (IQR)	
bone height at center	Baseline	13.8±2.8	15(5)	14.2±3.1	15(5)	0.551
	6 months following Sx	5.7±2	5.5(3)	6.7±2.9	6(4)	0.034*∗*
	Difference	8.1±1.9	8(3)	7.5±1.8	7(3)	0.389
	*p* Value		0.005*∗*		0.005*∗*	

bone height at mid-buccal (BH)	Baseline	6.2±2.3	6(3)	6.1±1.7	6(2)	0.763
	6 months following Sx	5.4±2.3	5.5(3)	5.8±1.7	6(2)	0.157
	Difference	0.8±1	1(2)	0.3±1.1	0.5(1)	0.096
	*p* Value		0.046*∗*		0.429	

bone width at 3 mm sub-crestal	Baseline	3.4±1.4	3(1)	4.2±2	4(4)	0.071
	6 months following Sx	3.5±1.1	3(1)	4.6±1.5	4.5(2)	0.031*∗*
	Difference	-0.1±1.1	0(1)	-0.4±1.6	-0.5(2)	0.257
	*p* Value		0.783		0.389	

bone width at 5 mm sub-crestal	Baseline	3.3±1.3	3(2)	3.6±1.2	4(3)	0.366
	6 months following Sx	3.2±1.1	3(2)	3.7±1	3.5(2)	0.132
	Difference	0.1±0.3	0(0)	-0.1±0.5	0(0)	0.317
	*p* Value		0.317		0.564	

Keratinized tissue width (KTW)	Baseline	3.7±1.2	4(2)	3.4±1.2	3.5(2)	0.18
	6 months following Sx	2±0.9	2(1)	3.4±1.2	3.5(2)	0.011*∗*
	Difference	1.7±0.6	2(1)	0±0	0(0)	0.004*∗*
	*p* Value		0.004*∗*		0.946	

Pain (VAS scale 0-10)	24 hours following Sx	3±0.8	3(2)	1.1±0.5	1(0)	0.006*∗*
	2 weeks following Sx	0.8±0.9	1(1)	0.4±0.5	0(1)	0.132
	Difference	2.2±1.2	1(1)	0.7±0.4	0(1)	0.023*∗*
	*p* Value		0.006*∗*		0.008*∗*	

*∗*p value <0.05

Note. CFT: closed flap technique, OFT: open flap technique, Sx: surgery.

## Data Availability

The clinical data used to support the findings of this study are restricted by the Institutional Review Board at Tufts Medical Center in order to protect patient privacy.
